# Network Meta-Analysis of Efficacy and Safety of Chemotherapy and Target Therapy in the First-Line Setting of Advanced Pancreatic Cancer

**DOI:** 10.3390/cancers11111746

**Published:** 2019-11-07

**Authors:** Kun-I Lin, Jia-Lian Yang, Yu-Chao Lin, Che-Yi Chou, Jin-Hua Chen, Chin-Chuan Hung

**Affiliations:** 1 Department of Obstetrics and Gynecology, Chang Bing Show Chwan Memorial Hospital, Changhua 500, Taiwan; kunidg107@gmail.com; 2 Department of Cosmetic Science, Providence University, Taichung 404, Taiwan; 3 Department of Pharmacy, College of Pharmacy, China Medical University, Taichung 404, Taiwan; u9903101@gmail.com; 4 Graduate Institute of Clinical Medical Science, China Medical University, Taichung 404, Taiwan; cmchlyc@yahoo.com.tw; 5 Division of Pulmonary and Critical Care Medicine, Department of Internal Medicine, China Medical University Hospital, Taichung 404, Taiwan; 6 Division of Nephrology, Asia University Hospital, Taichung 404, Taiwan; cychou.chou@gmail.com; 7 Department of Post-baccalaureate Veterinary Medicine, Asia University, Taichung 404, Taiwan; 8 Graduate Institute of Data Science, College of Management, Taipei Medical University, Taipei 110, Taiwan; jh_chen@tmu.edu.tw; 9 Department of Pharmacy, China Medical University Hospital, Taichung 404, Taiwan

**Keywords:** advanced pancreatic cancer, target therapy, chemotherapy, efficacy outcomes, adverse effects

## Abstract

Both gemcitabine and fluoropyrimidine are recommended backbones in the first-line treatment of pancreatic ductal adenocarcinoma (PDAC). To compare the efficacy and safety of these two therapeutic backbones, and to investigate the optimal therapies, we conducted a network meta-analysis. By retrospective analysis of randomized controlled trials (RCT), the most preferred therapeutic regimen may be predicted. The eligible RCTs of the gemcitabine-based therapies and fluoropyrimidine-based therapies were searched up to 31 August 2019. In a frequentist network meta-analysis, treatments were compared and ranked according to overall survival (OS) and progression-free survival (PFS). Thirty-two trials with 10,729 patients were included. The network meta-analyses results for overall survival and progression-free survival showed that fluoropyrimidine-based therapy seems to be the most effective treatment choice. Compared to gemcitabine combined with taxanes or immunotherapy, fluoropyrimidine-based therapy had comparable treatment effects (PFS: 0.67, *p*-Value = 0.11; 0.76, *p*-Value = 0.32; OS: 0.80, *p*-Value = 0.16; 0.77, *p*-Value = 0.21). Moreover, the combination of immunotherapy and gemcitabine had tolerable toxicities. Based on current evidence, fluoropyrimidine-based therapies and the combination of gemcitabine and taxanes were the most effective therapies in the advanced pancreatic cancer, and the combination of immunotherapy and gemcitabine can be developed into a new form of therapy.

## 1. Introduction

Pancreatic cancer is the fourth most common cause of cancer-related mortality worldwide. Patients with pancreatic ductal adenocarcinoma (PDAC) constituted almost 95% of pancreatic cancer patients, and 80% of PDAC patients were diagnosed with unresectable locally advanced or metastatic cancer due to the absence of specific symptoms in the early stages. Advanced PDAC patients had a poor prognosis with a five-year survival rate of 3% [1]. The vast majority of patients with advanced PDAC was treated with conventional chemotherapy or radiotherapy, with only modest survival benefits [2,3]. Therefore, developing novel anticancer mechanisms to reduce the mortality of patients with advanced PDAC is required.

Gemcitabine and fluoropyrimidine are the mainstream therapies for advanced PDAC treatment [2,3]. To improve patient survival rates, novel treatments combined with gemcitabine or fluoropyrimidine were recently developed. Because tyrosine kinase inhibitors achieve relatively useful responses in PDAC and their specific treatment effects produce relatively minor treatment-induced side effects, they have become a target to be developed [4]. The combination of gemcitabine and EGRF inhibitor, erlotinib, shows a statistical significance in prolonging median survival by two weeks relative to gemcitabine alone [5]. Moreover, gemcitabine combined with either tyrosine kinase inhibitors showed comparable efficacy and tolerable toxicities relative to gemcitabine [6,7]. Because PDAC has high microvascular density, antiangiogenetic measures can be applied to angiogenesis pathways; such therapies can block the pathways related to fibroblast growth factor, vascular endothelial growth factor, farnesyl-transferase, or the components of fibrotic tumor stroma [8,9,10]. High microvascular density, along with low microvessel integrity, was related to cancer progression, and targeting the angiogenesis may improve microvascular density, tumor perfusion, hypoxia reduction, and the efficiency of drug delivery [11]. Several meta-analyses have demonstrated that antiangiogenesis reduces the risk of progression in patients with advanced pancreatic cancer [12,13,14]. In addition to the combination of gemcitabine and targeted therapies, gemcitabine or fluoropyrimidine, combined with other chemotherapies, also significantly prolonged patient survival. A study proved that the combination of gemcitabine and taxanes increased the OS by two months [15]. Patients treated with fluorouracil, leucovorin, irinotecan, and oxaliplatin (FOLFIRINOX) obtained an increase in median survival of 4.3 months, but this treatment increased the risk of G3 to G5 hematologic toxicities [16]. Moreover, the role of immunity in PDAC remains unclear; the B- and T cells of PDAC patients can be induced to recognize some tumor-associated antigens, such as Wilms’ tumor gene 1 (WT1), mucin 1 (MUC1), human telomerase reverse transcriptase (hTERT), mutated K-RAS, and carcinoembryonic antigen (CEA). As antigen-specific immunity combined with the gemcitabine, gemcitabine can increase the expression of antigens to sensitize tumor cells to antigens [17,18]. The effects of gemcitabine is not limited to specific cytotoxic T-lymphocytes; it also modulates the immune functions, enhances antigen cross-presentation, and selectively inhibits the function of myeloid-derived suppressor cells. Another promising approach for fighting PDAC is to use bacteria to promote immunosurveillance [18]. In advanced PDAC, IMM-101 derived from heat-killed Mycobacterium obuense combined with gemcitabine can prolong median progression-free survival by nearly two months relative to gemcitabine alone [18].

Until now, no head-to-head trial has been done on the various gemcitabine-based therapies and fluoropyrimidine-based therapies that demonstrate antitumor potential for advanced PDAC; therefore, the optimal treatment remains to be identified. Network meta-analysis is a biostatistical method that can estimate relative effects from direct and indirect comparisons. To clarify the roles of new gemcitabine-based therapies or fluoropyrimidine-based therapies in the first line setting, this study performed a systematic review and a network meta-analysis to compare the efficacy and toxicity of gemcitabine-based therapies and fluoropyrimidine-based therapies.

## 2. Results

### 2.1. Search Results

**Figure 1:**
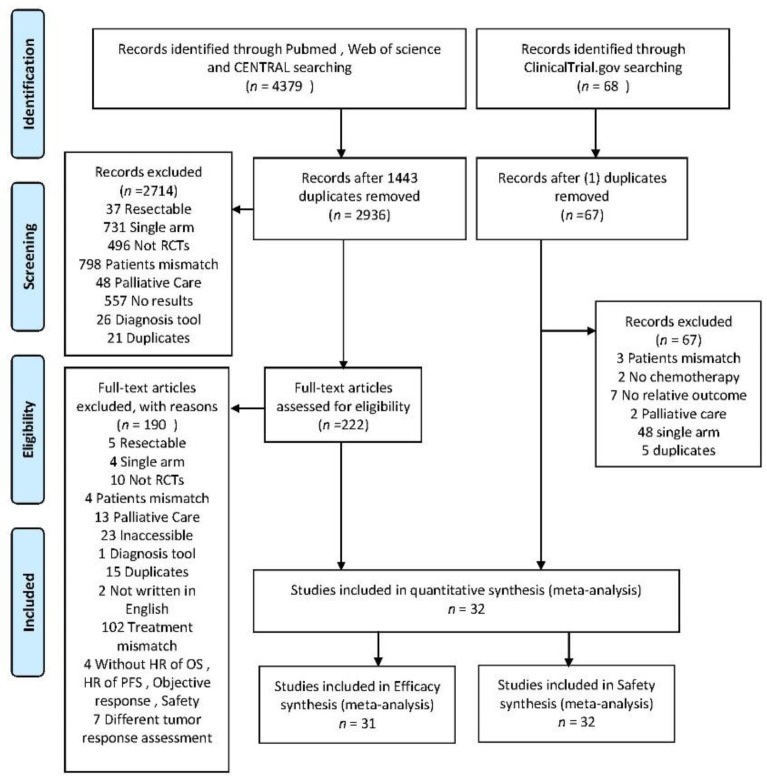
PRISMA flow diagram of randomized controlled trials included and excluded.

A total of 222 studies were evaluated by two independent reviewers and 32 RCTs involving 10,729 patients were included to conduct a meta-analysis and indirect comparisons (Figure 1). The detail characteristics of included trials are shown in Table S1. In order to reduce the heterogeneity, trials of immunotherapies limited to gemcitabine as a single arm are required. Among the included trials, gemcitabine was the common control group. Seven anticancer mechanisms were analyzed, including antiangiogenetic agents (bevacizumab, aflibercept, elpamotide, and vandetanib), multi-tyrosine kinase receptor inhibitors (axitinib, dasatinib, masitinib, sorafenib, and sunitinib), immune therapy (WT1 vaccine, and IMM-101), epidermal growth factor receptor (erlotinib, cetuximab, and nimotuzumab), taxanes (docetaxel, cationic liposomal paclitaxel, and nab-paclitaxel), platinum (oxaliplatin and cisplatin), fluoropyrimidine alone (S-1 and capecitabine), and fluoropyrimidine-based (FOLFIRINOX: folinic acid, fluorouracil, irinotecan, and oxaliplatin) (Figure S1) [5,6,7,15,16,19,20,21,22,23,24,25,26,27,28,29,30,31,32,33,34,35,36,37,38,39,40,41,42,43,44,45].

### 2.2. Risk of Bias

The quality of the included RCTs was generally good, with a low risk of bias (Figure S2). The most bias without blinding was observed in about 48% of included trials with open-label designed [15,21,23,24,25,26,27,28,30,33,36,39,40,42,45]. About 40% of included trial didn’t provide the detail information of allocation method [5,15,20,21,22,29,30,33,34,35,37,41,44,45]. One trial with unclear risk was due to coming from the meeting abstracts [22].

### 2.3. Overall Survival (OS)

The results of the pairwise meta-analysis of OS were presented in Figure S3. The combination of gemcitabine and fluoropyrimidine, combination of gemcitabine and taxanes, the combination of gemcitabine and taxanes, and fluoropyrimidine-based demonstrated the significantly survival benefit (HR: 0.86; 95% CI: 0.78–0.95; *p* = 0.002, heterogeneity: *p* = 0.59, *I*^2^ = 0%; HR: 0.71; 95% CI: 0.62–0.82; *p* < 0.00001, heterogeneity: *p* = 0.71, *I*^2^ = 0%; HR: 0.57; 95% CI: 0.46–0.70; *p* < 0.00001, heterogeneity: single study). Indirect comparisons performed to compare the OS was in Table 1. The combination of taxanes and gemcitabine and the fluoropyrimidine-based therapy had better survival benefit than most combination therapies, including the combination of antiangiogenesis and gemcitabine, the combination of EGFR inhibitors and gemcitabine, and the combination of fluoropyrimidine and gemcitabine. The use of the fluoropyrimidine-based therapy significantly improved 36% mortality risk (HR: 0.64; 95% CI: 0.47–0.86) as compared to the combination of platinum and gemcitabine. Though the use of fluoropyrimidine-based therapy showed a trend of better survival benefit as compared to the use of a combination of immunotherapy and gemcitabine (HR: 0.77; 95% CI: 0.51–1.16) and the use of a combination of taxanes and gemcitabine (HR: 0.80; 95% CI: 0.59–1.10), there was no significant difference.

### 2.4. Progression-Free Survival (PFS)

The results of the pairwise meta-analysis of PFS was presented in Figure S4. Statistically significant improvement of PFS were shown in the combination of fluoropyrimidine and gemcitabine (HR: 0.75; 95% CI: 0.65–0.86; *p* < 0.0001; heterogeneity: *p* = 0.13; *I*^2^ = 46%), the combination of immunotherapy and gemcitabine (HR: 0.62; 95% CI: 0.44–0.86; *p* = 0.004; heterogeneity: *p* = 0.70; *I*^2^ = 0%), fluoropyrimidine-based therapies (HR: 0.47; 95% CI: 0.38–0.58; *p* < 0.00001; heterogeneity: single trial), as well as the combination of taxanes and gemcitabine (HR: 0.70; 95% CI: 0.59–0.82; *p* < 0.00001; heterogeneity: *p* = 0.76; *I*^2^ = 0%), versus gemcitabine alone. Indirect comparisons of hazards ratios of PFS were showed in the Table 1. Fluoropyrimidine-based therapies had superior effect on improving the risk of progression than most combination therapies. It is noticed that the fluoropyrimidine-based therapies demonstrated the comparable effects with the combination of immunotherapy and gemcitabine, as well as the combination of taxes and gemcitabine (HR: 0.76; 95% CI: 0.44–1.31; HR: 0.67; 95% CI: 0.42–1.07). In addition, there was no significant difference between the combination of immunotherapy and gemcitabine and the combination of taxes and gemcitabine (HR: 0.87; 95% CI: 0.54–1.43).

### 2.5. Objective Response Rate (ORR)

The result of the pairwise meta-analysis of ORR is shown in Figure S5. Statistically significant increasing of ORR were shown in the combination of fluoropyrimidine and gemcitabine (RR: 2.00; 95% CI: 1.57–2.54; *p* < 0.0001; heterogeneity: *p* = 0.35; *I*^2^ = 11%), the use of fluoropyrimidine-based therapies (RR: 3.38; 95% CI: 2.01–5.65; *p* < 0.00001; heterogeneity: single study), and the use of fluoropyrimidine alone (RR: 1.64; 95% CI: 1.09–2.47; *p* = 0.002; heterogeneity: single study), versus gemcitabine alone. The indirect comparison analysis of ORR showed similar results to the indirect comparison of the hazard ratio of OS that the use of the fluoropyrimidine-based therapies could have a better objective response rate than most therapies (Table 2). Still, fluoropyrimidine-based therapies demonstrated the comparable effects with the combination of immunotherapy and gemcitabine, as well as the combination of taxes and gemcitabine (RR: 2.09; 95% CI: 0.60–7.22; RR: 1.55; 95% CI: 0.68–3.54). No inconsistency was observed in this outcome (Table S2).

### 2.6. Toxicity

**Figure 2:**
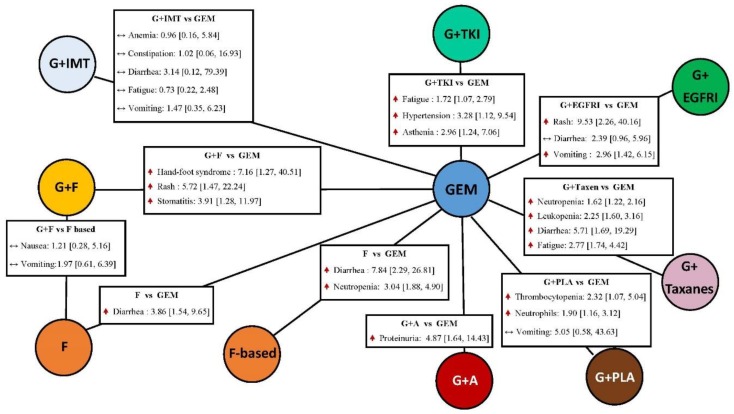
Pairwise comparisons of toxicities: experimental therapy vs. gemcitabine alone. Data presented as odds ratio (OR) with 95% confidence interval (CI); the 95% confidence interval that did not contain the value of 1 represents as statistical significance. PLA: doublet platinum-based treatment; GEM: gemcitabine; F: fluoropyrimidine only; F-based: fluoropyrimidine-based treatment; EGFRI: epidermal growth factor receptor inhibitor; ANGI: angiogenesis inhibitor; IMT: immunotherapy; TKI: tyrosine kinase inhibitor.

The statistically significant results of adverse events with grade 3 to 5 were shown in Figure 2. In the hematological toxicities, as compared to gemcitabine alone, fluoropyrimidine-based increased the risk the neutropenia (OR: 3.04; 95% CI: 1.88–4.90; *p* < 0.00001; heterogeneity: single trial). Adding platinum drugs to gemcitabine can increase the risk of thrombocytopenia (OR: 2.32; 95% CI: 1.07–5.04; *p* =0.03; heterogeneity: single trial) and abnormal neutrophils counts (OR: 1.90; 95% CI: 1.16–3.12; *p* = 0.01; heterogeneity: single trial) when compared to gemcitabine alone. On the other hand, patients treated with combination of taxanes and gemcitabine had higher neutropenia (OR: 1.62; 95% CI: 1.22–2.16; *p* = 0.00009; heterogeneity: *I*^2^ = 0%) and leukopenia (OR: 2.25; 95% CI: 1.60–3.16; *p* < 0.00001; heterogeneity: single trial). In the gastrointestinal disorders, the combination of EGFR inhibitor and gemcitabine was more toxic compared to gemcitabine alone, with the increased risk of vomiting (OR: 2.96; 95% CI: 1.42–6.15; *p* = 0.004; heterogeneity: *I*^2^ = 0%). Moreover, increasing risks of diarrhea were observed in the use of the combination of taxanes and gemcitabine, fluoropyrimidine alone, and fluoropyrimidine-based (OR: 5.71; 95% CI: 1.69–19.29; *p* = 0.005; heterogeneity: *I*^2^ = 0%; OR: 3.86; 95% CI: 1.54–9.65; heterogeneity: *I^2^* = 12%; OR: 0.84, 95% CI: 2.29–26.81; heterogeneity: single trial), as compared to gemcitabine alone. A higher risk of stomatitis was found in the combination of fluoropyrimidine and gemcitabine than in the gemcitabine alone (OR: 3.91; 95% CI: 1.28–11.97; *p* = 0.02; heterogeneity: *I*^2^ = 0%). With regard to the skin disorders, when compared to gemcitabine alone, the higher risk was observed in the combination fluoropyrimidine and gemcitabine, as well as in the combination of EGFR inhibitors and gemcitabine. Hand-foot syndrome occurred most in the combination of fluoropyrimidine and gemcitabine (OR: 7.16; 95% CI: 1.27–40.51; *p* = 0.03; heterogeneity: *I*^2^ = 0%). In addition, the combination of antiangiogenesis and gemcitabine was associated with a greater risk of proteinuria as compared to gemcitabine alone (OR: 4.87; 95% CI: 1.64–14.43; *p* = 0.004; *I*^2^ = 0%). The indirect comparison results of the risk of all grade 3 to 5 adverse events presented in the Figure S6 indicates that the combination of TKIs and gemcitabine had higher risk than gemcitabine alone (OR: 2.07; 95% CI: 1.09–3.94) and the combination of fluoropyrimidine and gemcitabine (OR: 3.26; 95% CI: 1.20–8.90). On the other hand, compared to gemcitabine alone, patients treated with the combination of taxanes and gemcitabine had a significantly higher risk of severe toxicities (OR: 1.33; 95% CI: 1.01–1.75).

### 2.7. Publication Bias

No significant publication bias of primary outcomes (OS) was identified in Figure S7.

### 2.8. Finding of Ranking 

**Figure 3:**
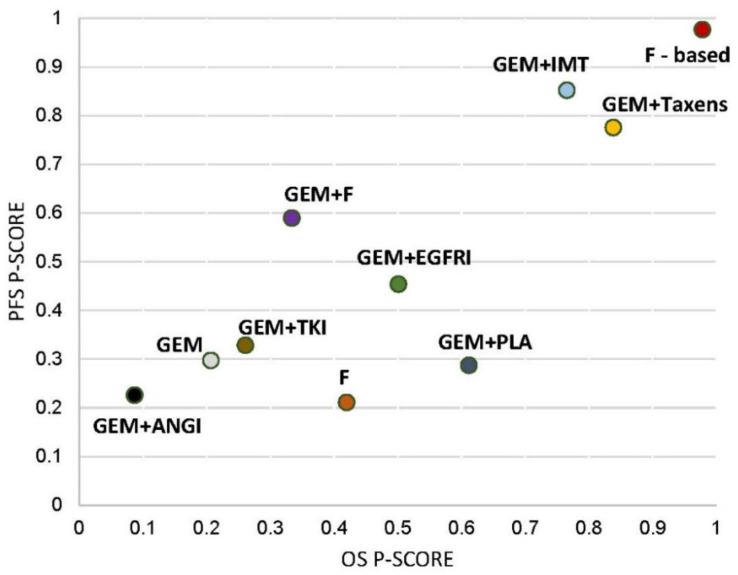
P-scores ranking plot. P-scores ranking plot represented the overall survival and the progression-free survival of interventions for advanced-staged pancreatic cancer. Treatment with best efficacy should be in the right-upper corner of the graph. PLA: doublet platinum-based treatment; GEM: gemcitabine; F: fluoropyrimidine only; F-based: fluoropyrimidine-based treatment; EGFRI: epidermal growth factor receptor inhibitor; ANGI: angiogenesis inhibitor; IMT: immunotherapy; TKI: tyrosine kinase inhibitor.

Ranking analysis for progression-free survival (PFS) performed with P-score suggested that the fluoropyrimidine-based was the best treatment option (P-score: 0.98), followed by the combination of immunotherapy and gemcitabine (P-score: 0.85), as well as the combination of taxanes and gemcitabine (P-score: 0.78) (Figure 3). In terms of ranking analysis for overall survival (OS), the fluoropyrimidine-based was the most efficacious (P score: 0.98) with the combination of taxanes and gemcitabine (P-score: 0.84), as well as the combination of immunotherapy and gemcitabine (P score: 0.77). The sum of ranking finding for overall survival (OS) and progression-free survival (PFS) was presented together in a bivariate ranking plot, which showed the most efficacious treatment in the upper-right corner of the graph (Figure 3). Conclusively, it suggested the fluoropyrimidine-based treatment demonstrated the best efficacy.

## 3. Discussion

Fluoropyrimidine-based and gemcitabine-based therapies are recommended as preferred first-line treatments for advanced PDAC [2,3]. The relative effects of different synergistic effects among the commonly used combination therapies were unknown. This network meta-analysis summarized direct and indirect comparisons of commonly used and newly implemented regimens to address this controversial issue.

The results confirmed previous observations [46,47,48,49] and suggested that fluoropyrimidine-based therapies and the combination of taxanes and gemcitabine had the best efficacy with different toxicity profiles in grade 3 to grade 5. The fluoropyrimidine-based therapies can be recommended in patients with higher risks of fatigue and leukopenia. In terms of patients with higher risks of diarrhea and neutropenia, the combination of taxanes and gemcitabine might be the treatment of choice. A combination of immunotherapy and gemcitabine demonstrated efficacy similar to that of the two aforementioned regimens but with lower toxicity. Synergistic effects of immunotherapy and chemotherapy can be proposed as follows: (a) exhaust immune suppressive cells; (b) enhance antibody-dependent cell-mediated cytotoxicity by releasing Ags expressed on tumor cells; and (c) activate helper and cytotoxic T cells. Any one of these mechanisms could strengthen the tumor-specific immune responses triggered by various immunotherapies [17,18]. The included immunotherapy trials were phase 2 clinical trials, which may exaggerate the treatment effects; nevertheless, a phase 2 trial is still the optimal approach to predict the effects of a novel treatment in a phase 3 trial. Some ongoing studies (NCT03611556, NCT01896869, NCT02826486, NCT03002831, NCT00044031, NCT00040092, NCT02362048, NCT01303172, NCT03509298, and NCT03983057) should be included in the future, for further investigation.

In this study, tyrosine kinase inhibitors did not significantly reduce mortality and progression, although such treatments initially showed high response rates and tolerable toxicities. Both KRAS/BRAF mutation and gene amplification may have contributed to resistance mechanisms. Nearly 90% of patients with PDAC possess KRAS mutations, and half of the remaining patients harbor BRAF mutations. KRAS/BRAF mutations cause increasing signaling of proliferation despite the inhibition of TKIs. Aside from KRAS/BRAF mutations, TKIs-treated tumors can gradually generate persistent genetic alterations and allow tumor toleration through compensatory pathways [4]. Our pairwise-analysis results of the combination of gemcitabine and antiangiogenesis did not present the benefit of improving progression, which may have mainly resulted from the different study design.

The present indirect comparison utilized the latest clinical data to assess potential mechanisms and combination therapies. Nevertheless, this study had several limitations. First, integrating different drugs with similar mechanisms in one arm may cause heterogeneity. In this study, however, most arms had low heterogeneity; high heterogeneity was only observed in the combination of EGFR inhibitors and gemcitabine. The limited extent of the heterogeneity may have been due to the characteristics of some patients enrolled in the LAP07 trial [39], which included more localized advanced patients. In the covariate analysis reported in the NCIC CTG Trial [5], patients with performance scores of two or with metastatic disease had relatively improved progression in the treatment arm, with the combination of erlotinib and gemcitabine. The bias of small sample size may overestimate the results of covariate analysis [50]. Two awkward situations (marginal benefit and the absence of biomarkers) were present that would require further trials for precise estimation of the patient groups that had truly benefited. A second limitation was that the results of all grade 3–5 results (Figure S6) did not contain any fluoropyrimidine-based therapies. Nevertheless, the results of pairwise results of adverse events (Figure 2) provided the distributions of general grade-3 to grade-5 toxicities in all included therapies. Third, because of the statistical requirement of indirect comparison, all trials without gemcitabine as comparison were excluded. Because most of the trials investigated immunotherapies (MUC-1, dendritic cells vaccine, telomerase, Cy/GVAX, and PD-1/PD-L1 inhibitors), and fluoropyrimidine-based therapies were not compared to gemcitabine alone, some bias may exist in the estimation of the treatment effects of immunotherapies and fluoropyrimidine-based therapies. To solve this problem, trials of immunotherapies were limited to the reference arm as gemcitabine alone in the present study, to reduce the heterogeneity. Thus, the role of the combination of immunotherapy and gemcitabine among the gemcitabine backbone therapies could be predicted. In terms of fluoropyrimidine-based therapies, benefits to survival and median survival with fluoropyrimidine-based therapies reported in the present study were similar to previous studies [51,52]. Therefore, the role and potential of fluoropyrimidine-based therapy in prolonging patient lifespan and reducing the risk of progression may still be estimated in the present study. As for the data showing the difference in terms of increased OS and PFS in weeks or months, we were not able to calculate from the included trials in the present study. Due to the heterogeneity of the different follow-up periods and patients of each included trial, we estimated the comparison results of overall survival and progression-free survival by using hazard ratio, which was adjusted for those variables [53].

Notwithstanding the limitations of this study, both the potential therapeutic efficacy and safety of the combination of immunotherapy (IMI-101 and WT1) and gemcitabine were observed in this network meta-analyses. Moreover, the results of the heterogeneity analysis and the inconsistency analysis validated the quality of relative effects (Table S2). These results can provide clinical information for developing first-line regimens for advanced PDAC patients.

## 4. Materials and Methods

### 4.1. Literature Search and Study Selection

Preferred Reporting Items for Systematic Reviews and Meta-Analyses for RCTs (Table S3) was followed, and the protocol was approved in the PROSPERO (Identifier: CRD42019128343).

A data search of randomized controlled trials of advanced pancreatic cancer and treatment published up to 31 August 2019, was performed on the Cochrane Central Register of Controlled Trials (CENTRAL), Web of science, EMBASE and Medline. The meeting abstracts from the American Society of Clinical Oncology (ASCO) and European Society for Medical Oncology (ESMO) were also searched for eligible trials. To search for unpublished data of randomized controlled trials, the US National Institutes of Health Ongoing Trials Register (Clinicaltrial.gov) was used. The following medical subject headings (MeSH) and text words for pancreatic cancer and treatments were used: pancreatic cancer (PDAC) AND gemcitabine OR cisplatin OR oxaliplatin OR carboplatin OR paclitaxel OR docetaxel OR irinotecan OR 5-FU OR S-1 AND clinical trial.

The inclusion criteria of eligible randomized controlled trials were as follows: (1) prospective randomized controlled trials reporting on efficacy and toxicity; (2) enrolled patients with unresectable locally advanced or metastatic pancreatic cancer; (3) the performance scores of enrolled patients were less than to 2 or KPS score higher than 50; (4) aged at least 18 years old, with adequate hematological, renal, and liver function; (5) the life expectancy of at least 8 weeks; (6) language limited to English or Chinese. To reduce the statistic bias of estimates of the treatment effects in the indirect comparison analysis, treatment of trial without gemcitabine alone would be excluded. Included studies were independently evaluated by two reviewers and reconciled with a third author if discrepancies occurred.

### 4.2. Data Extraction and Risk-of-Bias Assessment

The primary endpoint was overall survival (OS). The secondary endpoints were progression-free survival (PFS), objective response rate (ORR), and grade 3 to 5 adverse events (according to National Cancer Institute Common Terminology Criteria for Adverse Events version 4.0). The hazard ratios (HRs) with 95% confidence intervals (CIs) of PFS and OS were extracted. As for ORR and grade 3 to 5 adverse events, the number of patients and the number of total events were extracted for estimating. In addition, each patient’s performance score, dosing regimen, study design, and follow-up time were collected. As for the risk of bias of included trials, Cochrane risk-of-bias tool (version 5.1.0) was used to score the low, high, or unknown risk of bias, according to items. Two independent reviewers performed the data extraction and risk-of-bias assessment, and a third author handled with disagreement.

### 4.3. Statistical Analysis 

All analyses were performed with the frequentist model. For the efficacy evaluation, the outcome measures of overall survival and progression-free survival were the hazard ratios (HRs) with 95% CIs. For ORR, the outcome measures of response rate were risk ratios (RRs) with 95% CIs. In the outcome of toxicity, the outcome measures of grade 3 to 5 toxicities were odds ratios (ORs) with 95% CIs.

The direct meta-analysis, odds ratio, risk ratio, and hazard ratios were pooled by using a DerSimonian-Laird random-effects model in Revman 5.3. (Cochrane Collaboration, Copenhagen: The Nordic Cochrane Centre, The Cochrane Collaboration, 2014.). The random-effects model was based on the inverse-variance approach, which adjusts the study weight by considering the heterogeneity among the intervention effects. The Mantel–Haenszel method was used to calculate the odds ratio and risk ratio, and the inverse-variance method was used for estimating the hazard ratio. Next, an indirect comparison was done with the random-effects model in Frequentist framework [54,55]. Direct and indirect treatment effects were merged into a single effect size, and the relative effects between interventions were presented as ORs and HRs, with 95% CIs. The important assumption of indirect comparison is transitivity [56,57]. With this assumption, the estimated effect of intervention A versus intervention B can be obtained via intervention C, if the information of intervention A versus intervention C and intervention B versus intervention C were available [58]. The ORs, RRs, and HRs were pooled by STATA 13.1 (StataCorp, College Station, TX, USA) and R software 3.60 (R Core Team, (2013)). R: A language and environment for statistical computing. R Foundation for Statistical Computing, Vienna, Austria. URL http://www.R-project.org/) with netmeta package (https://CRAN.R-project.org/package=netmeta).

To evaluate the heterogeneity, Cochran’s Q test and the *I*² statistic were used to evaluate the heterogeneity across the studies. Heterogeneity existed with a *p*-value lower than 0.05 in Cochran’s Q test [59]. For network meta-analysis, node-spilt models were used to assess the inconsistency [60]. A funnel plot was used to assess the publication bias [61].

## 5. Conclusions

Overall, our results suggested that the combination of immunotherapy and gemcitabine could be developed as first-line therapies for advanced PDAC patients. On the other hand, fluoropyrimidine-based therapies and the combination of gemcitabine and taxanes were the most effective therapies in advanced pancreatic cancer. These findings enhanced our knowledge of the efficacy and safety of common combination therapies for advanced PDAC and increased our ability to identify the appropriate synergistic effects of combination therapies.

## Figures and Tables

**Table 1:** Indirect comparison of overall survival and progression free survival. Results of indirect comparison of overall survival are presented in the left lower half, and results from indirect comparison of progression-free survival are presented in the upper-right half.

PFS HR (95% CI) ^#^									
**F**	2.32 (1.39 to 3.88) *	1.09 (0.76 to 1.55)	1.03 (0.66 to 1.60)	1.17 (0.79 to 1.76)	1.27 (0.85 to 1.90)	1.77 (1.04 to 3.00) *	1.06 (0.63 to 1.76)	1.54 (0.98 to 2.45)	1.10 (0.72 to 1.68)
1.68 (1.20 to 2.35) *	**F-Based**	0.47 (0.32 to 0.68) *	0.44 (0.28 to0.70) *	0.51 (0.33 to 0.77) *	0.55 (0.36 to 0.83) *	0.76 (0.44 to 1.31)	0.46 (0.27 to 0.77) *	0.67 (0.41 to 1.07)	0.47 (0.31 to 0.73) *
0.96 (0.77 to 1.20)	0.57 (0.44 to 0.73) *	**GEM**	0.94 (0.72 to 1.23)	1.08 (0.89 to 1.30)	1.17 (0.97 to 1.41)	1.62 (1.09 to 2.40) *	0.97 (0.67 to 1.40)	1.42 (1.06 to 1.90) *	1.01 (0.80 to 1.27)
0.90 (0.69 to 1.17)	0.53 (0.40 to 0.71) *	0.94 (0.81 to 1.08)	**GEM + ANGI**	1.14 (0.82 to1.59)	1.24 (0.89 to 1.72)	1.72 (1.07 to 2.77) *	1.03 (0.65 to 1.62)	1.50 (1.01 to 2.24) *	1.07 (0.75 to 1.53)
1.03 (0.80 to 1.33)	0.61 (0.46 to0.81) *	1.08 (0.95 to 1.22)	1.15 (0.96 to 1.39)	**GEM + EGFRI**	1.08 (0.83 to 1.42)	1.50 (0.97 to 2.33)	0.90 (0.59 to 1.36)	1.32 (0.93 to 1.86)	0.93 (0.69 to 1.26)
1.13 (0.88 to 1.45)	0.67 (0.51 to 0.88) *	**1.17 (1.04 to 1.32)** *	1.25 (1.04 to 1.51) *	1.09 (0.92 to 1.29)	**GEM + F**	1.39 (0.89 to 2.15)	0.83 (0.55 to 1.26)	1.21 (0.85 to 1.72)	0.86 (0.64 to 1.16)
1.30 (0.88 to 1.92)	0.77 (0.51 to 1.16)	1.36 (0.98 to 1.87)	1.45 (1.02 to 2.06) *	1.26 (0.89 to 1.77)	1.16 (0.82 to 1.63)	**GEM + IMT**	0.60 (0.35 to 1.03)	0.87 (0.54 to 1.43)	0.62 (0.39 to 0.98) *
1.07 (0.81 to 1.42)	0.64 (0.47 to 0.86) *	1.12 (0.95 to 1.33)	1.20 (0.96 to 1.49)	1.04 (0.84 to 1.28)	0.95 (0.78 to 1.17)	0.83 (0.57 to 1.19)	**GEM + PLA**	1.46 (0.91 to 2.34)	1.04 (0.67 to 1.61)
**1.35 (1.01 to 1.80)** *	0.80 (0.59 to 1.10)	1.41 (1.17 to 1.69) *	1.50 (1.19 to 1.90) *	1.31 (1.05 to 1.63) *	1.20 (0.96 to 1.50)	1.04 (0.72 to 1.50)	1.26 (0.98 to 1.61)	**GEM + Taxanes**	0.71 (0.49 to 1.03)
0.97 (0.74 to 1.27)	0.58 (0.43 to 0.78) *	1.01 (0.87 to 1.18)	1.08 (0.88 to 1.33)	0.94 (0.77 to 1.15)	0.86 (0.71 to 1.05)	0.75 (0.52 to 1.07)	0.90 (0.72 to 1.14)	0.72 (0.57 to 0.92) *	**GEM + TKI**
**OS HR (95% CI) ^#^**									

^#^ Comparisons between treatments were read from left to right, and the estimate (hazard ratio, HR) with 95% confidence interval for a given comparison was read in the intersection of two treatments. In the left lower half and upper-right half, estimates with *p* < 0.05 favored the column-defining treatment. * Denotes *p*-value < 0.05. PLA: doublet platinum-based treatment; GEM: gemcitabine; F: fluoropyrimidine only; F-based: fluoropyrimidine-based treatment; EGFRI: epidermal growth factor receptor inhibitor; ANGI: angiogenesis inhibitor; IMT: immunotherapy; TKI: tyrosine kinase inhibition.

**Table 2:** Indirect comparison of objective response rate. Results of indirect comparison for objective response ratio were presented in the left lower half.

**F**									
0.42 (0.18 to 0.98) *	**F-based**								
1.40 (0.83 to 2.36)	3.37 (1.71 to 6.67) *	**GEM**							
1.15 (0.55 to 2.41)	2.76 (1.17 to 6.52) *	0.82 (0.48 to 1.38)	**GEM + ANGI**						
1.24 (0.64 to 2.40)	2.97 (1.34 to 6.62) *	0.88 (0.58 to 1.34)	1.08 (0.55 to 2.11)	**GEM + EGFRI**					
0.79 (0.48 to 1.32)	1.91 (0.91 to 4.00)	0.57 (0.42 to 0.75) *	0.69 (0.38 to 1.26)	0.64 (0.39 to 1.05)	**GEM + F**				
0.87 (0.27 to 2.77)	2.09 (0.60 to 7.22)	0.62 (0.22 to 1.74)	0.76 (0.24 to 2.42)	0.70 (0.23 to 2.15)	1.10 (0.37 to 3.21)	**GEM + IMT**			
0.93 (0.47 to 1.86)	2.25 (0.99 to5.09)	0.67 (0.42 to1.05)	0.81 (0.41 to 1.63)	0.76 (0.41 to 1.40)	1.18 (0.69 to 2.02)	1.08 (0.35 to 3.33)	**GEM + PLA**		
0.65 (0.32 to 1.30)	1.55 (0.68 to 3.54)	0.46 (0.29 to 0.73) *	0.56 (0.28 to 1.14)	0.52 (0.28 to 0.98) *	0.81 (0.47 to 1.41)	0.74 (0.24 to 2.32)	0.69 (0.38 to 1.27)	**GEM + Taxanes**	
0.90 (0.43 to 1.90)	2.17 (0.92 to 5.15)	0.64 (0.38 to 1.09)	0.79 (0.37 to 1.66)	0.73 (0.37 to 1.44)	1.14 (0.62 to 2.08)	1.04 (0.32 to 3.33)	0.97 (0.48 to 1.94)	1.40 (0.69 to 2.84)	**GEM + TKI**
**ORR RR (95% CI) ^#^**									

**^#^** Comparisons between treatments were read from left to right, and the estimate (risk ratio, RR) with a 95% confidence interval for a given comparison was read in the intersection of two treatments. The value of estimates higher than 1 indicated that column-defining treatment had better efficacy. * Denotes *p*-Value < 0.05. PLA: doublet platinum-based treatment; GEM: gemcitabine; F: fluoropyrimidine only; F-based: fluoropyrimidine-based treatment; EGFRI: epidermal growth factor receptor inhibitor; ANGI: angiogenesis inhibitor; IMT: immunotherapy; TKI: tyrosine kinase inhibitor. * *p* < 0.05. PLA: doublet platinum-based treatment; GEM: gemcitabine; F: fluoropyrimidine only; F-based: fluoropyrimidine-based treatment; EGFRI: epidermal growth factor receptor inhibitor; ANGI: angiogenesis inhibitor; IMT: immunotherapy; TKI: tyrosine kinase inhibitor.

## Supplementary Materials

The following are available online at https://www.mdpi.com/2072-6694/11/11/1746/s1. Table S1: Characteristics of included trials. Table S2: Consistency analyses of objective response rate by node-split model. Table S3: PRISMA NMA Checklist of Items to Include When Reporting a Systematic Review Involving a Network Meta-Analysis. Figure S1: Network plot for included therapies. Figure S2: Risk of bias. Figure S3: Pairwise comparisons of overall survival: experimental therapy vs. gemcitabine alone. Figure S4: Pairwise comparisons of progression-free survival: experimental therapy vs. gemcitabine alone. Figure S5: Pairwise comparisons of objective response rate: experimental therapy vs. gemcitabine alone. Figure S6: Forest plot of indirect comparison for all grade 3 to 5 adverse events. Figure S7: Funnel plot of overall survival.
