# Supplementary material for: Network Meta-Analysis of Efficacy and Safety of Chemotherapy and Target Therapy in the First-Line Setting of Advanced Pancreatic Cancer
Source: Cancers (Basel). 2019 Nov 7;11(11):1746. doi: 10.3390/cancers11111746 (PMC6895788; doi:10.3390/cancers11111746)
Supplement: Supplementary file 1 [file cancers-11-01746-s001.pdf]

Supplementary Materials:

Table S1: Characteristics of included trials

NCT number	Year	Author	phase	blind	muticenter	PS	Num	Treatment	Dose	follow up
					**GEM versus Fluropyrimidine only**					
NCT00498225	2013	H. Ueno	3	Open Label	muticenter	0 to 1	277	gemcitabine	1 g/m2 on days 1, 8, and 15 of a 28-day cycle.	18.4 months (0.3- 36.9)
							280	S-1	Orally twice daily at a dose according to the BSA on days through 28 of a 42-day cycle.	
							277	Gemcitabine + S-1	Gemcitabine at a dose of 1 g/m2 on days 1 and 8 plus S-1 orally twice daily at a dose according to the BSA on days 1 through 14 of a 21-day cycle.	
NCT00498225.	2017	T. Okusaka	3	open-label	muticenter	0 to 1	277	gemcitabine	1 g/m2 on days 1, 8, and 15 of a 28-day cycle.	29.8 months (0.3–46.3)
							280	S-1	Orally twice daily at a dose according to the BSA on days through 28 of a 42-day cycle.	
							275	Gemcitabine + S-1	Gemcitabine at a dose of 1 g/m2 on days 1 and 8 plus S-1 orally twice daily at a dose according to the BSA on days 1 through 14 of a 21-day cycle.	
					**GEM versus Fluropyrimidine based**					
NCT00112658.	211	Conroy	2/3	NA	muticenter	0 to 1	171	FOLFIRINOX	Oxaliplatin : 85 mg/BSA(m^2)<br>Irinotecan, 180 mg /BSA(m^2)<br>leucovorin, 400 mg /BSA(m^2)<br>fluorouracil, 400 mg /BSA(m^2) followed by<br>2400 mg /BSA(m^2) every 2 weeks	26.6 months
							171	gemcitabine	At a dose of 1000 mg/BSA(m^2) weekly for 7 of 8 weeks and then weekly for 3 of 4 weeks	

BSA: Body Surface Area

Table S1: Characteristics of included trials (continued)

Gemcitabine based versus Gemcitabine with Target therapies : GEM+ Fluoropyrimidine										
NCT number	Year	Author	phase	blind	muticenter	PS	Num	Treatment	Dose	follow up
Ref[34]	2007	R. Herrmann	3	NA	muticenter	KPS >= 60%	160	Gemcitabine + capecitabine	Capecitabine 650 mg/m2 BID days 1 to 14 every 3 weeks. Gemcitabine 1 g/m2 on days 1 and 8 every 3 weeks.	36 months
							159	Gemcitabine	1 g/m2 weekly for 7 weeks, followed by a 1-week break, and then weekly for 3 weeks every 4 weeks	
Ref[44]	2017	H. S. Lee	3	NA	muticenter	0 to 2	108	Gemcitabine + capecitabine	Capecitabine 1660mg/m2 daily for 3 weeks followed by a 1-week break plus Gemcitabine 1000mg/m2 weekly for 3 weeks every 4 weeks.	40 months
							106	Gemcitabine	1000mg/m2 weekly for 3 weeks every 4 weeks.	
Ref[25]	2012	Y. Nakai	2	Open Label	muticenter	0 to 2	53	Gemcitabine	1 g/m2 on days 1, 8, and 15 of each 4-week cycle.	24 months
							53	Gemcitabine + S-1	Gemcitabine 1 g/m2 on days 1 and 15 and S-1 BID for 2 weeks followed by a 2-week rest between each 4-week cycle.	
NCT00514163.	2012	M. Ozaka	2	Open Label	NA	0 to 2	59	Gemcitabine	1 g/m2 on days 1, 8, and 15, as 1 course repeated every 4 weeks.	36 months
							53	Gemcitabine + S-1	Gemcitabine 1 g/m2 on days 1 and 8, and 40 mg/m2 and S-1 taken BID on days 1–14, every 3 weeks.	
Ref[33]	2009	D. Cunningham	3	Open Label	muticenter	0 to 2	266	Gemcitabine	Gemcitabine at 1 g/m2 weekly x 7 , followed by 1 week rest, then weekly x 3 every 4 weeks.	27 months
							267	Gemcitabine + capecitabine	Gemcitabine at 1 g/m2 weekly x 3 every 4 weeks. Capecitabine at 1,660 mg/m2 daily for 3 weeks followed by 1 week's rest.	
UMIn000002244	2013	Sudo	3	Open Label	NA	0 to 1	51	Gemcitabine	1 g/m2 on days 1, 8 and 15 every 4 weeks	48 months
							50	Gemcitabine + S-1	S-1 at 60 mg/m2 divided in two daily doses on days 1–15 and Gemcitabine at 1 g/m2 on days 8 and 15 every 3 weeks	

Table S1 : Characteristics of included trials (continued)

NCT number	Year	Author	phase	blind	muticenter	PS	Num	Treatment	Dose	follow up
Ref[45]	2008	S. Boeck	2	Open Label	muticenter	KPS >= 60%	61	Capecitabine + oxaliplatin	Capecitabine 1 g/m2 BID on days 1–14 followed by 1-week rest Oxaliplatin 130 mg/m2 on day 1.	46.6months
							64	Gemcitabine + capecitabine	capecitabine 825 mg/m2 t mg/m2 BID days 1–14 followed by 1-week rest Gemcitabine 1 g/m2 on day 1 and 8.	
							63	Gemcitabine + oxaliplatin	Gemcitabine 1 g/m2 on day 1 and 8. Oxaliplatin 130 mg/m2 on day 8.	
**Gemcitabine based versus Gemcitabine with Target therapies : GEM+Anti-angiogenesis**										
Ref[35]	2010	H. L. Kindler	3	Double-blind	muticenter	0 to 2	302	Gemcitabine + bevacizumab	Gemcitabine at 1,000mg/m2was on days 1, 8, and 15 of a 28-day cycle. Bevacizumab at 10 mg/kg after gemcitabine on days 1 and 15 of each cycle.	50 months
							300	Gemcitabine	1,000mg/m2 on days 1, 8, and 15 of a 28-day cycle	
Ref[31]	2013	P. Rougier	3	Double-blind	muticenter	0 to 2	271	Gemcitabine + aflibercept	Aflibercept 4 mg/kg every 2 weeks on days 1 and 15 of every 4-week cycle Gemcitabine 1 g/m2 on days 1, 8, 15 and 22 of cycle 1 and then days 1, 8 and 15 of subsequent 28-day cycles	7.8 months.
							275	Gemcitabine + placebo	1 g/m2 on days 1, 8, 15 and 22 of cycle 1 and then days 1, 8 and 15 of subsequent 28-day cycles	
Ref[29]	2015	H. Yamaue	2/3	Double-blind	muticenter	0 to 1	100	Elpamotide + gemcitabine	Gemcitabine (1 g/m2) on days 1, 8, and 15 as one cycle every 4 weeks. Elpamotide (2.0 mg ⁄mL⁄ body) every week	33 months
							53	Gemcitabine + Placebo	Gemcitabine (1 g/m2) on days 1, 8, and 15 as one cycle every 4 weeks. Placebo (1.0 mL⁄ body)	

Table S1: Characteristics of included trials (continued)

NCT number	Year	Author	phase	blind	muticenter	PS	Num	Treatment	Dose	follow up
ISRCTN 96397434.	2017	G. Middleton	2	Double-blind	muticenter	0 to 1	72	Vandetanib + Gemcitabine	Gemcitabine 1 g/m2 weekly for 7 weeks followed by a 1-week break. After this period, gemcitabine 3 weeks followed by a 1-week break. Vandetanib was once daily at 300 mg/day	Median 24·9 months
							70	Gemcitabine	Gemcitabine 1 g/m2 weekly for 7 weeks followed by a 1-week break. After this period, gemcitabine 3 weeks followed by a 1-week break.	
**Gemcitabine based versus Gemcitabine with Target therapies : GEM+EGFRI**										
Ref[5]	2007	M. J. Moore	3	Double-blind	muticenter	0 to 2	285	Gemcitabine + Erlotinib	Gemcitabine 1 g/m2 on days 1, 8, 15, 22, 29, 36, and 43 followed by a 1-week rest in cycle one (8 weeks), and on days 1, 8 and 15 in all subsequent 4-week cycles. Erlotinib 100 or 150 mg/d until disease progression or unmanageable toxicity.	24 months
							284	Gemcitabine	Gemcitabine 1 g/m2 on days 1, 8, 15, 22, 29, 36, and 43 followed by a 1-week rest in cycle one (8 weeks), and on days 1, 8 and 15 in all subsequent 4-week cycles.	
Ref[23]	2017	B. Schultheis	2b	Open Label	muticenter	KPS >= 70%	93	Gemcitabine +nimotuzumab	Gemcitabine 1 g/m2 once weekly for 3 weeks, followed by a 1-week rest; and l nimotuzumab 400 mg fixed dose weekly	12 months
							93	gemcitabine	Gemcitabine 1 g/m2 once weekly for 3 weeks, followed by a 1-week rest	

Table S1: Characteristics of included trials (continued)

NCT number	Year	Author	phase	blind	muticenter	PS	Num	Treatment	Dose	follow up
NCT00075686	2010	P. A. Philip	3	Open Label	muticenter	0 to 2	331	Gemcitabine	1 g/m2 .During the first 8 weeks, gemcitabine was administered weekly for 7 weeks followed by 1 week off. In all remaining cycles, gemcitabine was administered for 3 weeks followed by a week of rest.	48 months
							329	Gemcitabine + Cetuximab	Gemcitabine at a dose of 1 g/m2 .During the first 8 weeks, gemcitabine was administered weekly for 7 weeks followed by 1 week off. In all remaining cycles, gemcitabine administered for 3 weeks followed by a week of rest.<br>Cetuximab at a loading dose of 400 mg/m2 (over 120 minutes) on week 1, followed by weekly maintenance doses of 250 mg/m2 (over 60 minutes).	
NCT00634725.	2016	P. Hammel	3	Open Label	muticenter	0 to 2	223	gemcitabine	1g /m2 weekly for 3 weeks, followed by a 1-week rest (1 cycle), for 4 cycles.	33 months
							219	Gemcitabine + Erlotinib	Gemcitabine at a dose of 1g/m2 weekly for 3 weeks, followed by a 1-week rest (1 cycle), for 4 cycles.<br>Erlotinib 100mg QD	
Ref[22]	2016	P. Khatri (abstract)		NA	NA		36	Gemcitabine + Capecitabine	Gemcitabine 1 g/m2 as day 1and 8 every 3week, 30-minute infusion and capecitabine 850mg/m2 twice daily for 2 weeks with one week rest	
							30	Gemcitabine + Erlotinib	Gemcitabine 1 g/m2 as a 100-minute on day 1 and Erlotinib100mg daily	

Table S1: Characteristics of included trials (continued)

NCT number	Year	Author	phase	blind	muticenter	PS	Num	Treatment	Dose	follow up
**Gemcitabine based versus Gemcitabine with Target therapies : GEM+ Immune therapy**										
NCT01303172	2016	A. G. Dalgleish	2	Open Label	muticenter	0 to 2	75	Gemcitabine + IMM-101	Gemcitabine 1 g/m2 for 3 weeks out of 4, with dose reductions allowed for toxicity. IMM-101 (0.1 ml of 10 mg/ml suspension)<br>IMM-101 was administered every 2 weeks for three doses followed by 4 weeks rest, then every 2 weeks for a further three doses. Subsequent doses were administered every 4 weeks; the first IMM-101 dose was administered 2 weeks before the first dose of GEM.	12 months
							35	Gemcitabine	Gemcitabine 1 g/m2 weekly for 3 weeks out of 4	
UMIN 000005248	2018	S. Nishida	2	Open Label	muticenter	KPS >= 80%	42	Gemcitabine + WT1 vaccine	Gemcitabine 1 g/m2 on days 1, 8, and 15 of a 28-day cycle.<br>WT1 vaccine on day 1 and 15 of a 28-day cycle.	24 months
							43	gemcitabine	Gemcitabine 1 g/m2 intravenously on days 1, 8, and 15 of a 28-day cycle	
**Gemcitabine based versus Gemcitabine with Target therapies : GEM+TKI**										
NCT00541021	2012	A. Goncalves	3	Double-blind	muticenter	0 to 2	52	Gemcitabine + sorafenib	Gemcitabine 1 g/m2 on days 1, 8, 15, 22, 29, 36 and 42, with rest on day 49 (for cycle 1) and on days 1, 8, 15, 29, 36 and 42 with rest on days 22 and 49 (cycles 2 and 3) of 8-week cycles.<br>Sorafenib 400 mg BID continuously	median 27.7 months
							52	gemcitabine	Gemcitabine 1 g/m2 on days 1, 8, 15, 22, 29, 36 and 42, with rest on day 49 (for cycle 1) and on days 1, 8, 15, 29, 36 and 42 with rest on days 22 and 49 (cycles 2 and 3) of 8-week cycles.<br>Two tablets of placebo were given twice daily.	

Table S1 : Characteristics of included trials (continued)

NCT number	Year	Author	phase	blind	muticenter	PS	Num	Treatment	Dose	follow up
NCT00789633.	2015	G. Deplanque	3	Double-blind	muticenter	0 to 1	173	Gemcitabine + masitinib	Masitinib (9 mg/kg/day) was administered orally BID. Gemcitabine 1 g/m2 according to standard practice.	24 months
							176	gemcitabine	Gemcitabine 1 g/m2 according to standard practice.	
NCT00219557	2008	J. P. Spano	2	Open Label	muticenter	0 to 2	69	Gemcitabine +axitinib	axitinib 5 mg BID gemcitabine 1 g/m2 on days 1, 8, and 15 in 4-week cycles	18 months
							34	gemcitabine	gemcitabine 1 g/m2 on days 1, 8, and 15 in 4-week cycles	
NCT00471146	2011	H. L. Kindler	3	Double-blind	muticenter	0 to 1	314	Gemcitabine +axitinib	gemcitabine 1 g/m2 on days 1, 8, and 15 in 4-week cycles until disease progression axitinib 5 mg BID until disease progression,	27·4 weeks (0·1–55·5)
							316	gemcitabine	1 g/m2 on days 1, 8, and 15 in 4-week cycles until disease progression	
Ref[30]	2015	L. Bergmann	2	Open Label	muticenter	0 to 1	54	gemcitabine	1 g/m2 on days 1, 8 and 15 of a 28 day cycle.	29.25 moths
							52	Gemcitabine + sunitinib	sunitinib 50 mg/day in cycles of 3 weeks with a 2 weeks on/1 week off schedule.Gemcitabine 1 g/m2 on days 1, 8 and 15 of a 28 day cycle	
NCT01395017	2017	Evans TRJ	2	Double-blind	NA	0 to 1	100	Gemcitabine + Dasatinib	Gemcitabine 1 g/m2 weekly for 3 weeks of a 4-week cycle). Dasatinib 100 mg by mouth once daily (QD).	48 months
							102	Gemcitabine	Gemcitabine 1 g/m2 weekly for 3 weeks of a 4-week cycle)	

Table S1: Characteristics of included trials (continued)

NCT number	Year	Author	phase	blind	muticenter	PS	Num	Treatment	Dose	follow up
**Gemcitabine based versus Gemcitabine with Chemotherapy : Gemcitabine+Platin-based**										
Ref[37]	2009	E. Poplin	3	NA	NA	0 to 2	275	Gemcitabine	Gemcitabine 1 g/m2 weekly for 7 weeks followed by 1 week of rest; for the subsequent cycles, patients received cycles of Gemcitabine 1 g/m2 weekly for 3 weeks followed by 1 week rest	36moth
							272	Gemcitabine + oxaliplatin	Gemcitabine 1 g/m2 day 1. Oxaliplatin 100 mg/m2 day 2 every 14 days cycle	
NCT00813696	2010	G. Colucci	3	Open Label	muticenter	KPS >= 50%	199	Gemcitabine	1 g/m2 weekly for 7 weeks (cycle 1), followed by 1 week of rest. Gemcitabine was continued on days 1, 8, and 15 every 28 days.	median 38.2 months
							201	Gemcitabine +Cisplatin	Gemcitabine 1 g/m2 weekly for 7 weeks (cycle 1), followed by 1 week of rest. Gemcitabine was continued on days 1, 8, and 15 every 28 days.<br>Cisplatin 25 mg/m2 on days 1, 8, 15, 29, 36, and 42 of cycle 1, followed by 1 week of rest, Cisplatin on days 1, 8, and 15 every 28 days.	
**Gemcitabine based versus Gemcitabine with Chemotherapy : Gemcitabine+Taxanes**										
Ref[44]	2009	M. H. Kulke	2	NA	muticenter	0 to 2	62	Gemcitabine +Cisplatin	Gemcitabine 1 g/m2 on days, 1, 8, and 15, every 28 days.<br>Cisplatin 50 mg/m2 on days 1 and 15, every 28 days.	50 months
							58	Gemcitabine	Gemcitabine 1.5 g/m2 on days 1, 8, and 15, every 28 days.	
							65	Gemcitabine + Docetaxel	Gemcitabine 1 g/m2 on days 1 and 8, every 21 days.<br>Docetaxel 40 mg/m2 on days 1 and 8.	

Table S1: Characteristics of included trials (continued)

NCT number	Year	Author	phase	blind	muticenter	PS	Num	Treatment	Dose	follow up
Ref[21]	2012	J. M. Lohr	2	Open Label	muticenter	0 to 2	50	Gemcitabine	7 weekly Gemcitabine 1 g/m2 on days 4, 11, etc. and, if applicable, 14 twice-weekly infusions of ET on days 1, 4, etc. (10 min at 0.5 ml/min, 10 min at 1.0 ml/min and thereafter 1.5 ml/min)	28 months
							50	Gemcitabine + Liposomal paclitaxel	7 weekly Gemcitabine 1 g/m2 on days 4, 11. 14 twice-weekly infusions of liposomal paclitaxel 44 mg/m2 on days 1, 4, etc.	
NCT00844649	2013	D.D.Von Hoff	3	Open Label	muticenter	KPS >= 70%	431	Gemcitabine + nab-paclitaxel	nab-paclitaxel 125 mg/m2, followed by gemcitabine 1 g/m2on days 1, 8, 15, 29, 36, and 43	9.1 months (0.1 to 36.9)
							430	Gemcitabine	Gemcitabine 1 g/m2 weekly for 7 of 8 weeks .Subsequent, Gemcitabine on days 1, 8, and 15 every 4 weeks	7.4 months (0.0 to 31.3)

Table S2: Consistency analyses of objective response rate by node-split model

Comparison	Direct	Indirect	z	p-value
F vs. GEM	0.47	-0.06	0.87	0.3847
F vs. GEM+F	-0.32	0.32	-0.87	0.3847
GEM vs. GEM+EGFRI	-0.12	-0.2	0.12	0.9065
GEM vs. GEM+F	-0.57	-0.5	-0.12	0.9065
GEM vs. GEM+PLA	-0.33	-1.79	1.37	0.1704
GEM vs. GEM+Taxanes	-0.79	-0.6	-0.18	0.8606
GEM+EGFRI vs. GEM+F	-0.38	-0.46	0.12	0.9065
GEM+PLA vs. GEM+Taxanes	0.05	-0.56	0.91	0.3625

p-value less than 0.05 represented presence of inconsistency.

Table S3: PRISMA NMA Checklist of Items to Include When Reporting A Systematic Review Involving a Network Meta-analysis

Section/Topic	Item #	Checklist Item	Reported on Page #
**TITLE**			
Title	1	Identify the report as a systematic review *incorporating a network meta-analysis (or related form of meta-analysis).*	***P.1***
**ABSTRACT**			
Structured summary	2	Provide a structured summary including, as applicable:<br>**Background:** main objectives<br>**Methods:** data sources; study eligibility criteria, participants, and interventions; study appraisal; and *synthesis methods, such as network meta-analysis.*<br>**Results:** number of studies and participants identified; summary estimates with corresponding confidence/credible intervals; *treatment rankings may also be discussed. Authors may choose to summarize pairwise comparisons against a chosen treatment included in their analyses for brevity.*<br>**Discussion/Conclusions:** limitations; conclusions and implications of findings.<br>**Other:** primary source of funding; systematic review registration number with registry name.	***P.1***
**INTRODUCTION**			
Rationale	3	Describe the rationale for the review in the context of what is already known*, including mention of why a network meta-analysis has been conducted.*_	***P.2-3***
Objectives	4	Provide an explicit statement of questions being addressed, with reference to participants, interventions, comparisons, outcomes, and study design (PICOS).	***P.2-3***
**METHODS**			
Protocol and registration	5	Indicate whether a review protocol exists and if and where it can be accessed (e.g., Web address); and, if available, provide registration information, including registration number.	***P.10***
Eligibility criteria	6	Specify study characteristics (e.g., PICOS, length of follow-up) and report characteristics (e.g., years considered, language, publication status) used as criteria for eligibility, giving rationale. *Clearly describe eligible treatments included in the treatment network, and note whether any have been clustered or merged into the same node (with justification).*_	***P.10***
Information sources	7	Describe all information sources (e.g., databases with dates of coverage, contact with study authors to identify additional studies)	***P.10***

		in the search and date last searched.	
Search	8	Present full electronic search strategy for at least one database, including any limits used, such that it could be repeated.	***P.10***
Study selection	9	State the process for selecting studies (i.e., screening, eligibility, included in systematic review, and, if applicable, included in the meta-analysis).	***P.10***
Data collection process	10	Describe method of data extraction from reports (e.g., piloted forms, independently, in duplicate) and any processes for obtaining and confirming data from investigators.	***P.10***
Data items	11	List and define all variables for which data were sought (e.g., PICOS, funding sources) and any assumptions and simplifications made.	***P.10***
**Geometry of the network**	**S1**	Describe methods used to explore the geometry of the treatment network under study and potential biases related to it. This should include how the evidence base has been graphically summarized for presentation, and what characteristics were compiled and used to describe the evidence base to readers.	***P.10***
Risk of bias within individual studies	12	Describe methods used for assessing risk of bias of individual studies (including specification of whether this was done at the study or outcome level), and how this information is to be used in any data synthesis.	***P.10***
Summary measures	13	State the principal summary measures (e.g., risk ratio, difference in means). *Also describe the use of additional summary measures assessed, such as treatment rankings and surface under the cumulative ranking curve (SUCRA) values, as well as modified approaches used to present summary findings from meta-analyses.*	***P.10-11***
Planned methods of analysis	14	Describe the methods of handling data and combining results of studies for each network meta-analysis. This should include, but not be limited to:<br>• *Handling of multi-arm trials;*<br>• *Selection of variance structure;*<br>• *Selection of prior distributions in Bayesian analyses; and*<br>• *Assessment of model fit.*	***P.10-11***
**Assessment of Inconsistency**	**S2**	Describe the statistical methods used to evaluate the agreement of direct and indirect evidence in the treatment network(s) studied. Describe efforts taken to address its presence when found.	***P.10-11***
Risk of bias across studies	15	Specify any assessment of risk of bias that may affect the cumulative evidence (e.g., publication bias, selective reporting within studies).	***P.10***
Additional analyses	16	Describe methods of additional analyses if done, indicating which were pre-specified. This may include, but not be limited to, the following:	***P.10-11***

		• Sensitivity or subgroup analyses;<br>• Meta-regression analyses;<br>• *Alternative formulations of the treatment network; and*<br>• *Use of alternative prior distributions for Bayesian analyses (if applicable).*_	
**RESULTS†**			
Study selection	17	Give numbers of studies screened, assessed for eligibility, and included in the review, with reasons for exclusions at each stage, ideally with a flow diagram.	***P.3***<br>***Figure 1***
**Presentation of network structure**	**S3**	Provide a network graph of the included studies to enable visualization of the geometry of the treatment network.	***Figure S1***
**Summary of network geometry**	**S4**	Provide a brief overview of characteristics of the treatment network. This may include commentary on the abundance of trials and randomized patients for the different interventions and pairwise comparisons in the network, gaps of evidence in the treatment network, and potential biases reflected by the network structure.	***P.3-4***<br>***Table S1***
Study characteristics	18	For each study, present characteristics for which data were extracted (e.g., study size, PICOS, follow-up period) and provide the citations.	***P.3-4***<br>***Table S1***
Risk of bias within studies	19	Present data on risk of bias of each study and, if available, any outcome level assessment.	***P.4***<br>***Figure S2***
Results of individual studies	20	For all outcomes considered (benefits or harms), present, for each study: 1) simple summary data for each intervention group, and 2) effect estimates and confidence intervals. *Modified approaches may be needed to deal with information from larger networks.*	***P.4,5,6***<br>***Figure S3,S4,S5***
Synthesis of results	21	Present results of each meta-analysis done, including confidence/credible intervals. *In larger networks, authors may focus on comparisons versus a particular comparator (e.g. placebo or standard care), with full findings presented in an appendix. League tables and forest plots may be considered to summarize pairwise comparisons.* If additional summary measures were explored (such as treatment rankings), these should also be presented.	***Table 1 , 2***<br>***Figure 2***<br>***P. 4,5,6,7***<br>***Figure S3,S4,S5,S6***
**Exploration for inconsistency**	**S5**	Describe results from investigations of inconsistency. This may include such information as measures of model fit to compare consistency and inconsistency models, *P* values from statistical tests, or summary of inconsistency estimates from different parts of the treatment network.	***P.9***<br>***Table S2***
Risk of bias across studies	22	Present results of any assessment of risk of bias across studies for the evidence base being studied.	***P.4***<br>***Figure S2***
Results of	23	Give results of additional analyses, if done (e.g., sensitivity or	***P.7-8***

additional analyses		subgroup analyses, meta-regression analyses, *alternative network geometries studied, alternative choice of prior distributions for Bayesian analyses,* and so forth).	***Figure 3*** ***Figure S7.***
**DISCUSSION**			
Summary of evidence	24	Summarize the main findings, including the strength of evidence for each main outcome; consider their relevance to key groups (e.g., healthcare providers, users, and policy-makers).	***P.8,9***
Limitations	25	Discuss limitations at study and outcome level (e.g., risk of bias), and at review level (e.g., incomplete retrieval of identified research, reporting bias). *Comment on the validity of the assumptions, such as transitivity and consistency. Comment on any concerns regarding network geometry (e.g., avoidance of certain comparisons).*	***P.9***
Conclusions	26	Provide a general interpretation of the results in the context of other evidence, and implications for future research.	***P.11***
**FUNDING**			
Funding	27	Describe sources of funding for the systematic review and other support (e.g., supply of data); role of funders for the systematic review. This should also include information regarding whether funding has been received from manufacturers of treatments in the network and/or whether some of the authors are content experts with professional conflicts of interest that could affect use of treatments in the network.	***P.11***

(a) Overall survival

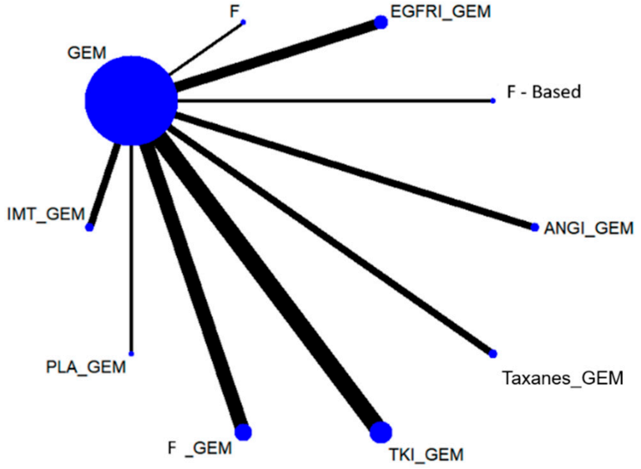

(b)Progression free survival

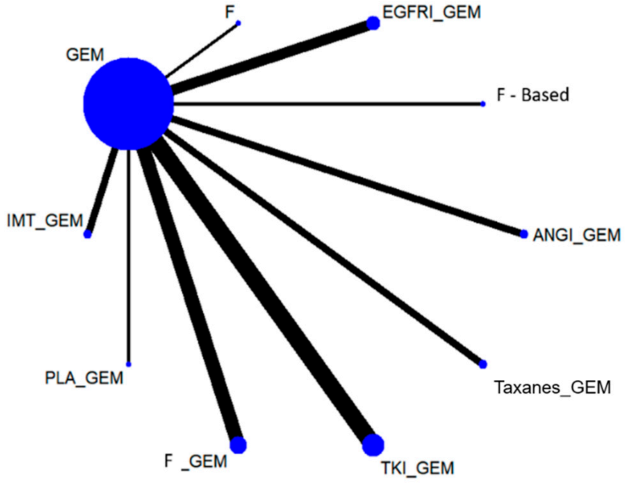

(c)Objective response rate

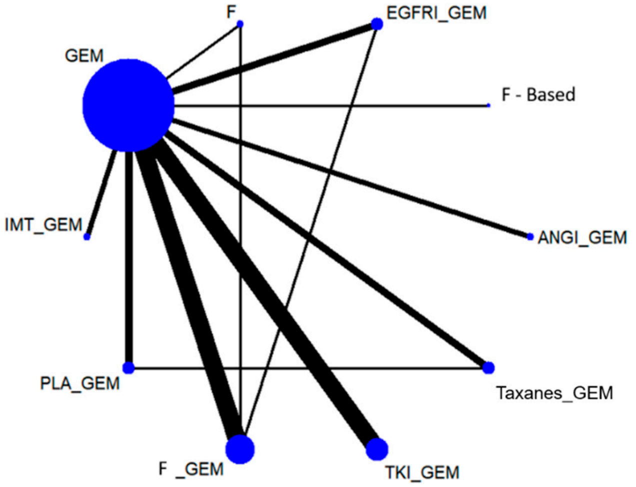

Figure S1: Network plot for included therapies.

The solid line represents as the direct comparisons. PLA: doublet platinum-based treatment; GEM: gemcitabine; F: fluoropyrimidine only; F - Based: fluoropyrimidine-based treatment; EGFRI: epidermal growth factor receptor inhibitor; ANGI: angiogenesis inhibitor; IMT: immunotherapy; TKI: tyrosine kinase inhibitor.

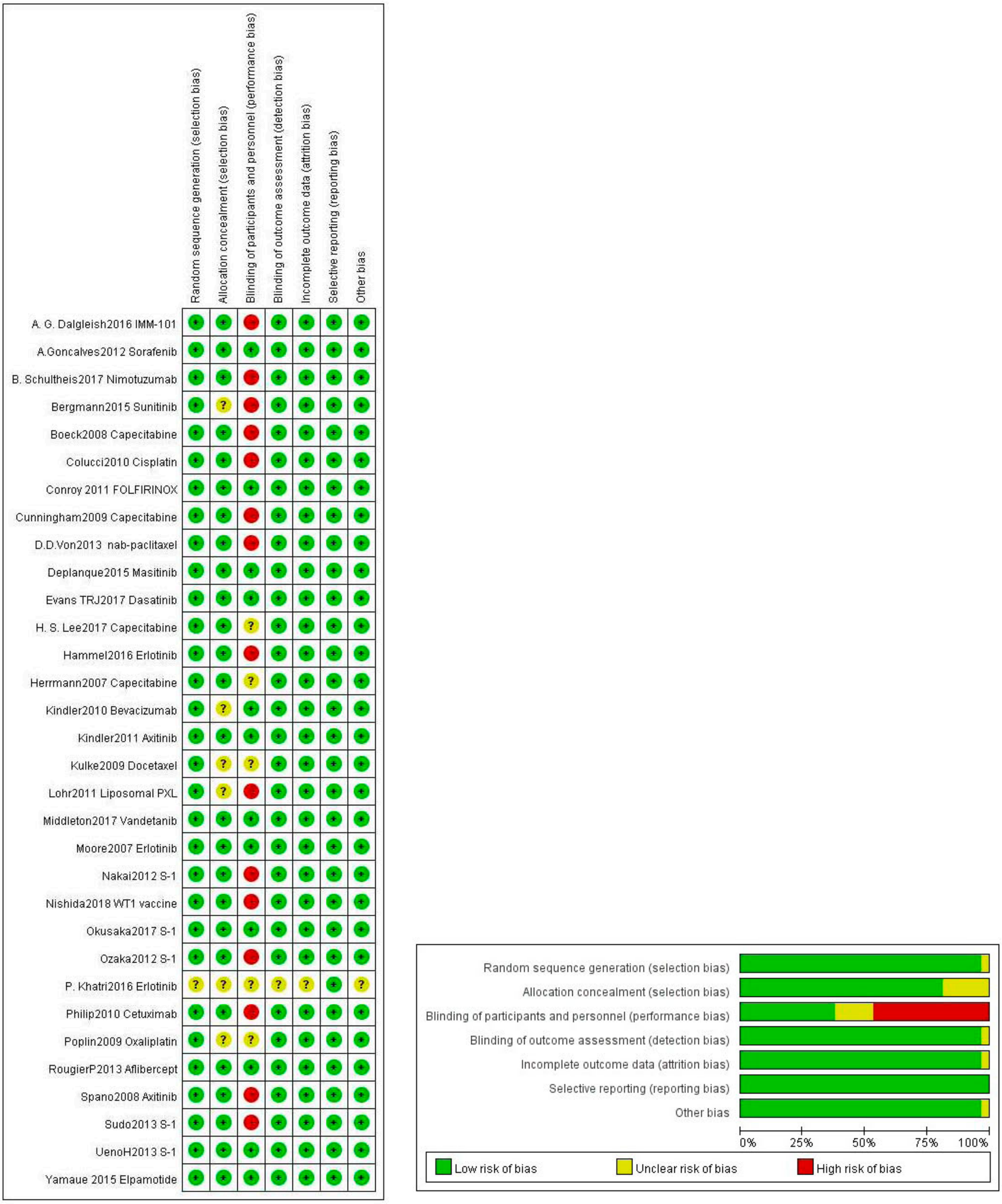

(a) (b)

Figure S2: Risk of bias. (a) Risk of bias summary: The judgements about each risk of bias item for each included study. (b) Risk of bias graph: The judgements about each risk of bias item reflected as percentages across all included studies.

Study or Subgroup	log[Hazard Ratio]	SE	Weight	Hazard Ratio IV, Random, 95% CI
**1.1.1 GEM+Fluoropyrimidine**				
Cunningham2009 Capecitabine	-0.1508	0.0871	5.3%	0.86 [0.73, 1.02]
H. S. Lee2017 Capecitabine	-0.19845094	0.10470099	4.7%	0.82 [0.67, 1.01]
Okusaka2017 S-1	-0.09431068	0.0875094	5.2%	0.91 [0.77, 1.08]
Ozaka2012 S-1	-0.462	0.2192	2.2%	0.63 [0.41, 0.97]
Sudo2013 S-1	-0.07257069	0.18702813	2.7%	0.93 [0.64, 1.34]
**Subtotal (95% CI)**			**20.0%**	**0.86 [0.78, 0.95]**
Heterogeneity: Tau² = 0.00; Chi² = 2.81, df = 4 (P = 0.59); I² = 0%				
Test for overall effect: Z = 3.09 (P = 0.002)				
**1.1.2 GEM+Anti-angiogenesis**				
Kindler2010 Bevacizumab	-0.04305949	0.0874585	5.2%	0.96 [0.81, 1.14]
Middleton2017 Vandetanib	0.19062036	0.12157169	4.2%	1.21 [0.95, 1.54]
RougierP2013 Aflibercept	0.15272109	0.119795	4.2%	1.16 [0.92, 1.47]
Yamaue 2015 Elpamotide	-0.13926207	0.29701723	1.4%	0.87 [0.49, 1.56]
**Subtotal (95% CI)**			**15.0%**	**1.07 [0.93, 1.22]**
Heterogeneity: Tau² = 0.00; Chi² = 3.59, df = 3 (P = 0.31); I² = 16%				
Test for overall effect: Z = 0.94 (P = 0.35)				
**1.1.3 GEM+EGFRI**				
B. Schultheis2017 Nimotuzumab	-0.3711	0.1746	2.9%	0.69 [0.49, 0.97]
Hammel2016 Erlotinib	0.17395331	0.10255683	4.8%	1.19 [0.97, 1.45]
Moore2007 Erlotinib	-0.19845	0.092095	5.1%	0.82 [0.68, 0.98]
Philip2010 Cetuximab	-0.05826891	0.07686858	5.6%	0.94 [0.81, 1.10]
**Subtotal (95% CI)**			**18.3%**	**0.91 [0.75, 1.11]**
Heterogeneity: Tau² = 0.03; Chi² = 10.60, df = 3 (P = 0.01); I² = 72%				
Test for overall effect: Z = 0.91 (P = 0.36)				
**1.1.4 GEM+ Immune therapy**				
A. G. Dalgleish2016 IMM-101	-0.38566248	0.2194391	2.2%	0.68 [0.44, 1.05]
Nishida2018 WT1 vaccine	-0.22314355	0.22116515	2.1%	0.80 [0.52, 1.23]
**Subtotal (95% CI)**			**4.3%**	**0.74 [0.54, 1.00]**
Heterogeneity: Tau² = 0.00; Chi² = 0.27, df = 1 (P = 0.60); I² = 0%				
Test for overall effect: Z = 1.96 (P = 0.05)				
**1.1.5 GEM+TKI**				
A.Goncalves2012 Sorafenib	0.2390169	0.21338953	2.2%	1.27 [0.84, 1.93]
Deplanque2015 Masitinib	-0.11653382	0.12216647	4.2%	0.89 [0.70, 1.13]
Evans TRJ2017 Dasatinib	0.14842	0.1815	2.8%	1.16 [0.81, 1.66]
Kindler2011 Axitinib	0.0139029	0.13011785	3.9%	1.01 [0.79, 1.31]
Spano2008 Axitinib	-0.34249031	0.24061178	1.9%	0.71 [0.44, 1.14]
**Subtotal (95% CI)**			**15.0%**	**0.99 [0.84, 1.16]**
Heterogeneity: Tau² = 0.01; Chi² = 4.82, df = 4 (P = 0.31); I² = 17%				
Test for overall effect: Z = 0.16 (P = 0.88)				
**1.1.6 Fluropyrimidine based**				
Conroy 2011 FOLFIRINOX	-0.56211892	0.10799039	4.6%	0.57 [0.46, 0.70]
**Subtotal (95% CI)**			**4.6%**	**0.57 [0.46, 0.70]**
Heterogeneity: Not applicable				
Test for overall effect: Z = 5.21 (P < 0.00001)				
**1.1.7 Fluropyrimidine**				
Okusaka2017 S-1	-0.040822	0.08766207	5.2%	0.96 [0.81, 1.14]
**Subtotal (95% CI)**			**5.2%**	**0.96 [0.81, 1.14]**
Heterogeneity: Not applicable				
Test for overall effect: Z = 0.47 (P = 0.64)				
**1.1.8 GEM+Platin**				
Colucci2010 Cisplatin	-0.0953	0.106285	4.6%	0.91 [0.74, 1.12]
Poplin2009 Oxaliplatin	-0.12783337	0.09272982	5.1%	0.88 [0.73, 1.06]
**Subtotal (95% CI)**			**9.7%**	**0.89 [0.78, 1.02]**
Heterogeneity: Tau² = 0.00; Chi² = 0.05, df = 1 (P = 0.82); I² = 0%				
Test for overall effect: Z = 1.63 (P = 0.10)				
**1.1.9 GEM+Taxanes**				
D.D.Von2013 nab-paclitaxel	-0.32850407	0.07441485	5.7%	0.72 [0.62, 0.83]
Lohr2011 Liposomal PXL	-0.41551544	0.222839	2.1%	0.66 [0.43, 1.02]
**Subtotal (95% CI)**			**7.8%**	**0.71 [0.62, 0.82]**
Heterogeneity: Tau² = 0.00; Chi² = 0.14, df = 1 (P = 0.71); I² = 0%				
Test for overall effect: Z = 4.78 (P < 0.00001)				
**Total (95% CI)**			**100.0%**	**0.89 [0.82, 0.96]**
Heterogeneity: Tau² = 0.02; Chi² = 63.28, df = 25 (P < 0.0001); I² = 60%				
Test for overall effect: Z = 3.12 (P = 0.002)				
Test for subgroup differences: Chi² = 37.46, df = 8 (P < 0.00001), I² = 78.6%				

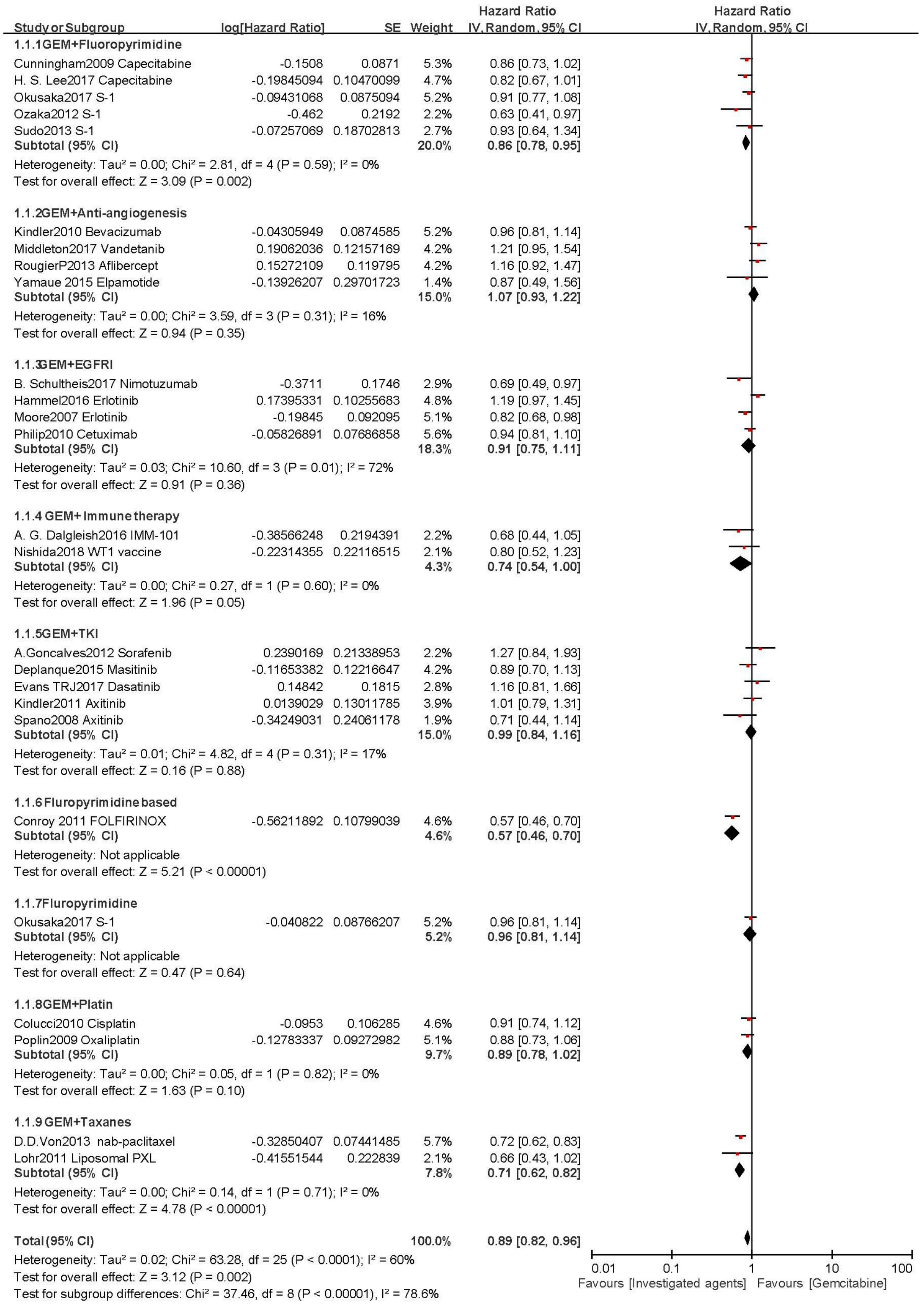

Figure S3: Pairwise comparisons of overall survival: experimental therapy vs. gemcitabine alone.

Data presented as hazard ratio (HR) with 95% confidence interval (CI); $p < 0.05$: statistically significance. Square represented estimated HR in each study and its size reflected the sample size; 95% CIs represented horizon lines; summary HR presented as diamond. The significant level of heterogeneity was $p < 0.05$. PLA: doublet platinum-based treatment; GEM: gemcitabine; F: fluoropyrimidine only; F - Based: fluoropyrimidine-based treatment; EGFRI: epidermal growth factor receptor inhibitor; ANGI: angiogenesis inhibitor; IMT: immunotherapy; TKI: tyrosine kinase inhibitor.

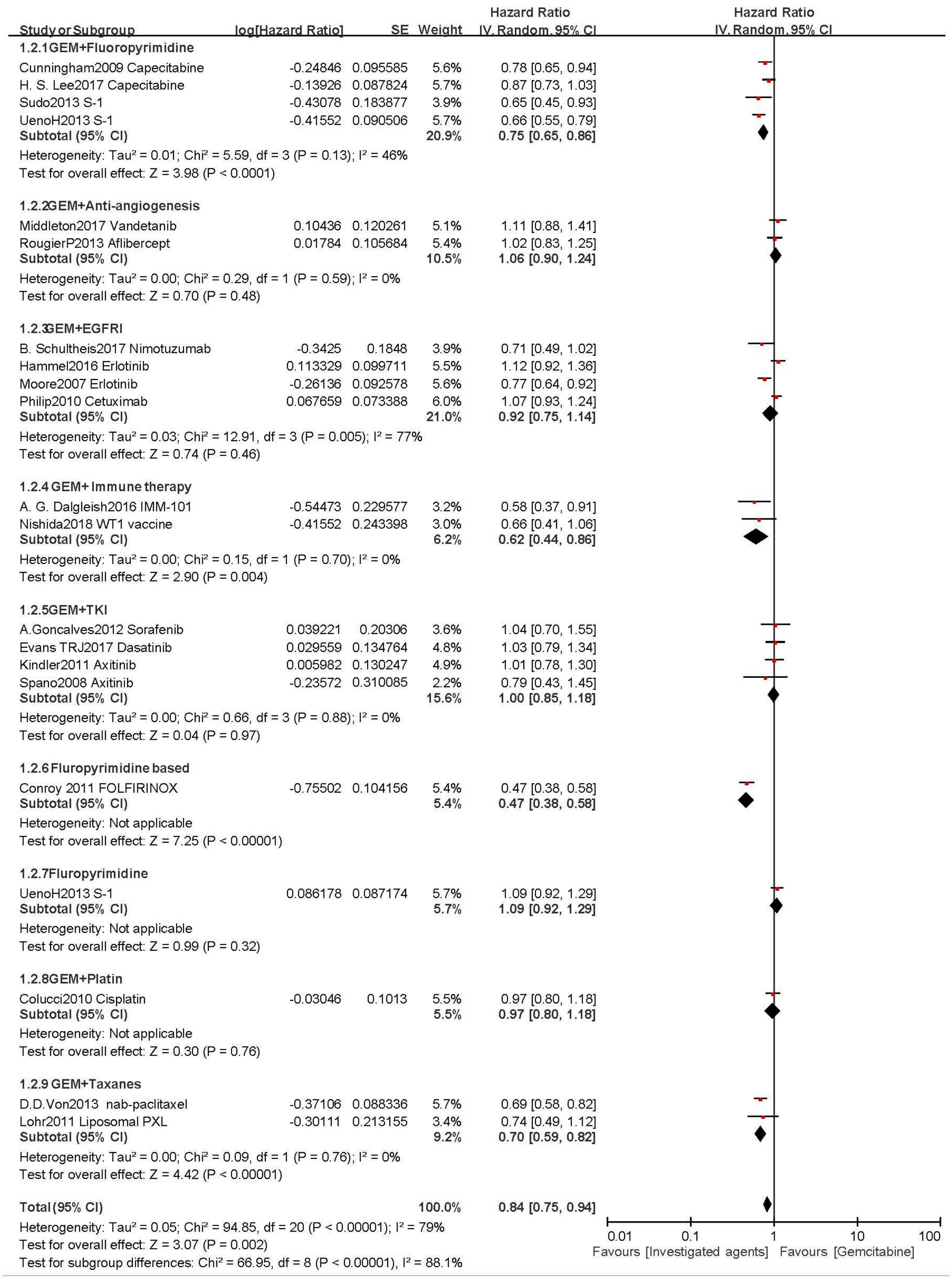

Figure S4: Pairwise comparisons of progression free survival: experimental therapy vs. gemcitabine alone.

Data presented as hazard ratio (HR) with 95% confidence interval (CI); $p < 0.05$: statistically significance. Square represented estimated HR in each study and its size reflected the sample size; 95% CIs represented horizon lines; summary HR presented as diamond. The significant level of heterogeneity was $p < 0.1$. PLA: doublet platinum-based treatment; GEM: gemcitabine; F: fluoropyrimidine only; F - Based: fluoropyrimidine-based treatment; EGFRI: epidermal growth factor receptor inhibitor; ANGI: angiogenesis inhibitor; IMT: immunotherapy; TKI: tyrosine kinase inhibitor.

Study or Subgroup	Investigated agents Events	Investigated agents Total	Gemcitabine Events	Gemcitabine Total	Weight	Risk Ratio M-H, Random, 95% CI
**1.3.1GEM+Fluoropyrimidine**						
Cunningham2009 Capecitabine	51	267	33	266	7.2%	1.54 [1.03, 2.31]
H. S. Lee2017 Capecitabine	38	108	16	106	5.7%	2.33 [1.39, 3.92]
Herrmann2007 Capecitabine	15	160	12	159	3.7%	1.24 [0.60, 2.57]
Nakai2012 S-1	10	53	5	53	2.3%	2.00 [0.73, 5.46]
Ozaka2012 S-1	15	53	4	59	2.2%	4.17 [1.48, 11.80]
Sudo2013 S-1	11	51	3	50	1.7%	3.59 [1.07, 12.12]
UenoH2013 S-1	71	277	32	277	7.5%	2.22 [1.51, 3.25]
**Subtotal (95% CI)**		**969**		**970**	**30.2%**	**2.00 [1.57, 2.54]**
Total events	211		105			
Heterogeneity: Tau² = 0.01; Chi² = 6.71, df = 6 (P = 0.35); I² = 11%						
Test for overall effect: Z = 5.68 (P < 0.00001)						
**1.3.2GEM+Anti-angiogenesis**						
Kindler2010 Bevacizumab	39	302	30	300	6.5%	1.29 [0.82, 2.02]
Middleton2017 Vandetanib	10	72	9	70	3.0%	1.08 [0.47, 2.50]
**Subtotal (95% CI)**		**374**		**370**	**9.6%**	**1.24 [0.84, 1.84]**
Total events	49		39			
Heterogeneity: Tau² = 0.00; Chi² = 0.14, df = 1 (P = 0.71); I² = 0%						
Test for overall effect: Z = 1.07 (P = 0.28)						
**1.3.3GEM+EGFRI**						
B. Schultheis2017 Nimotuzumab	8	93	8	93	2.6%	1.00 [0.39, 2.55]
Moore2007 Erlotinib	24	285	22	284	5.3%	1.09 [0.62, 1.89]
Philip2010 Cetuximab	28	329	23	331	5.5%	1.22 [0.72, 2.08]
**Subtotal (95% CI)**		**707**		**708**	**13.4%**	**1.13 [0.79, 1.62]**
Total events	60		53			
Heterogeneity: Tau² = 0.00; Chi² = 0.17, df = 2 (P = 0.92); I² = 0%						
Test for overall effect: Z = 0.69 (P = 0.49)						
**1.3.4 GEM+ Immune therapy**						
A. G. Dalgleish2016 IMM-101	8	75	1	35	0.7%	3.73 [0.49, 28.71]
Nishida2018 WT1 vaccine	6	42	5	43	2.0%	1.23 [0.41, 3.72]
**Subtotal (95% CI)**		**117**		**78**	**2.6%**	**1.58 [0.60, 4.19]**
Total events	14		6			
Heterogeneity: Tau² = 0.00; Chi² = 0.93, df = 1 (P = 0.33); I² = 0%						
Test for overall effect: Z = 0.92 (P = 0.36)						
**1.3.5GEM+TKI**						
A.Goncalves2012 Sorafenib	12	52	10	52	3.6%	1.20 [0.57, 2.53]
Bergmann2015 Sunitinib	2	52	2	54	0.7%	1.04 [0.15, 7.10]
Evans TRJ2017 Dasatinib	11	100	8	102	2.9%	1.40 [0.59, 3.34]
Kindler2011 Axitinib	12	314	4	316	1.9%	3.02 [0.98, 9.26]
Spano2008 Axitinib	5	69	1	34	0.6%	2.46 [0.30, 20.27]
**Subtotal (95% CI)**		**587**		**558**	**9.8%**	**1.53 [0.95, 2.46]**
Total events	42		25			
Heterogeneity: Tau² = 0.00; Chi² = 2.25, df = 4 (P = 0.69); I² = 0%						
Test for overall effect: Z = 1.74 (P = 0.08)						
**1.3.6 Fluropyrimidine based**						
Conroy 2011 FOLFIRINOX	54	171	16	171	5.7%	3.38 [2.01, 5.65]
**Subtotal (95% CI)**		**171**		**171**	**5.7%**	**3.38 [2.01, 5.65]**
Total events	54		16			
Heterogeneity: Not applicable						
Test for overall effect: Z = 4.62 (P < 0.00001)						
**1.3.7Fluropyrimidine**						
UenoH2013 S-1	52	277	32	280	7.1%	1.64 [1.09, 2.47]
**Subtotal (95% CI)**		**277**		**280**	**7.1%**	**1.64 [1.09, 2.47]**
Total events	52		32			
Heterogeneity: Not applicable						
Test for overall effect: Z = 2.38 (P = 0.02)						
**1.3.8GEM+Platin**						
Colucci2010 Cisplatin	26	201	20	199	5.3%	1.29 [0.74, 2.23]
Poplin2009 Oxaliplatin	27	272	16	275	4.8%	1.71 [0.94, 3.09]
**Subtotal (95% CI)**		**473**		**474**	**10.2%**	**1.47 [0.98, 2.19]**
Total events	53		36			
Heterogeneity: Tau² = 0.00; Chi² = 0.47, df = 1 (P = 0.49); I² = 0%						
Test for overall effect: Z = 1.85 (P = 0.06)						
**1.3.9 GEM+Taxanes**						
D.D.Von2013 nab-paclitaxel	99	431	31	430	7.5%	3.19 [2.18, 4.66]
Kulke2009 Docetaxel	7	65	6	58	2.2%	1.04 [0.37, 2.92]
Lohr2011 Liposomal PXL	5	50	5	50	1.8%	1.00 [0.31, 3.24]
**Subtotal (95% CI)**		**546**		**538**	**11.5%**	**1.69 [0.69, 4.13]**
Total events	111		42			
Heterogeneity: Tau² = 0.43; Chi² = 6.66, df = 2 (P = 0.04); I² = 70%						
Test for overall effect: Z = 1.16 (P = 0.25)						
**Total (95% CI)**		**4221**		**4147**	**100.0%**	**1.71 [1.45, 2.03]**
Total events	646		354			
Heterogeneity: Tau² = 0.06; Chi² = 39.44, df = 25 (P = 0.03); I² = 37%						
Test for overall effect: Z = 6.23 (P < 0.00001)						
Test for subgroup differences: Chi² = 16.60, df = 8 (P = 0.03), I² = 51.8%						

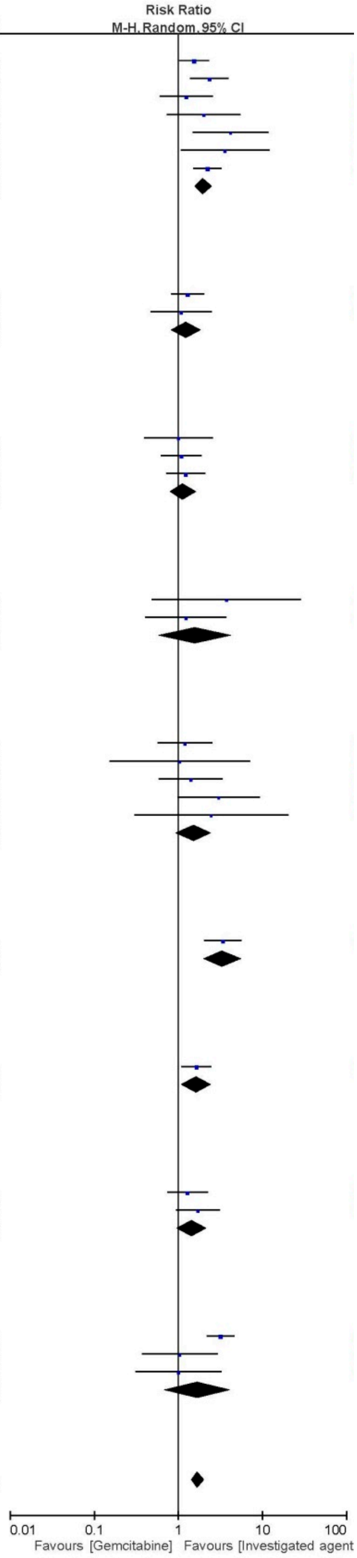

Figure S5: Pairwise comparisons of objective response rate : experimental therapy vs. gemcitabine alone.

Data presented as risk ratio (RR) with 95% confidence interval (CI); p < 0.05: statistically significance. Square represented estimated RR in each study and its size reflected the sample size; 95% CIs represented horizon lines; summary RR presented as diamond. The significant level of heterogeneity was p < 0.1. PLA: doublet platinum-based treatment; GEM: gemcitabine; F: fluoropyrimidine only; F - Based: fluoropyrimidine-based treatment; EGFRI: epidermal growth factor receptor inhibitor; ANGI: angiogenesis inhibitor; IMT: immunotherapy; TKI: tyrosine kinase inhibitor.

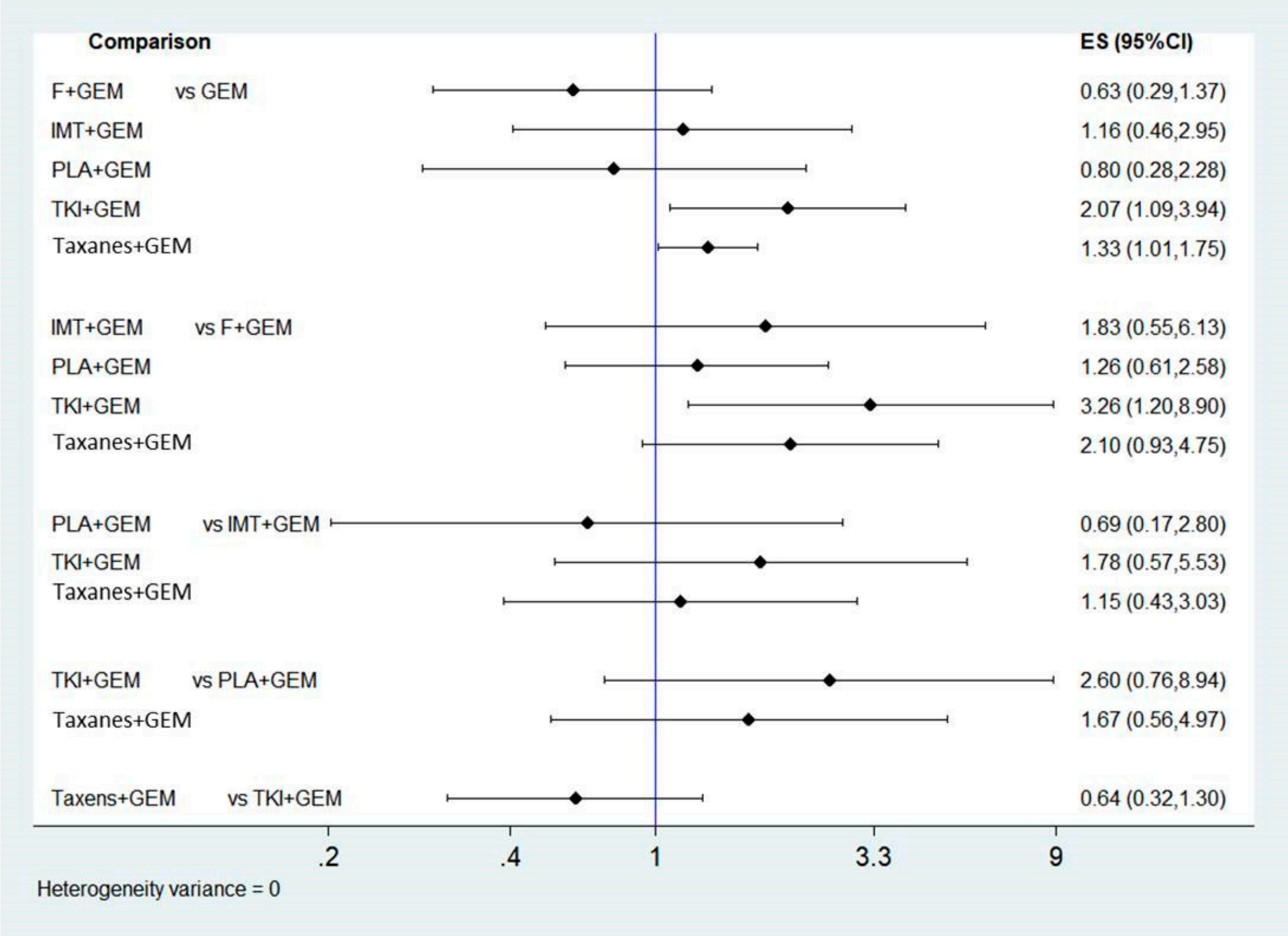

Figure S6: Forest plot of indirect comparison for all grade 3 to 5 adverse events.
All individual regimens compared with reference treatment. Odds ratios (OR) and 95% confidence intervals were given. . PLA: doublet platinum-based treatment; GEM: gemcitabine; F: fluoropyrimidine; EGFRI: epidermal growth factor receptor inhibitor; ANGI: angiogenesis inhibitor; IMT: immunotherapy; TKI: tyrosine kinase inhibitor.

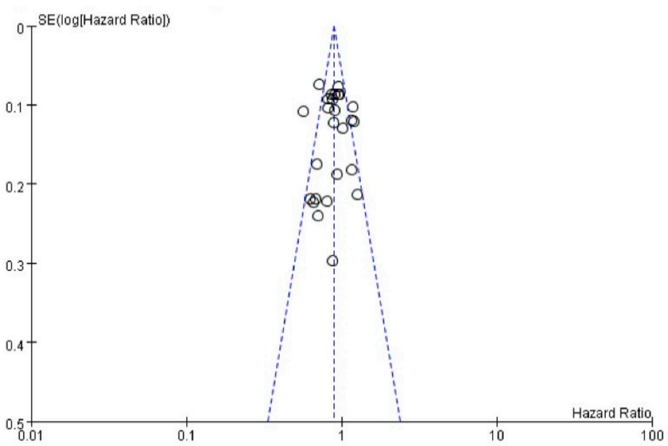

Figure S7: Funnel plot of overall survival

Each dot reflected a study; the vertical axis reflected the sample size or standard error and x-axis reflected the effect size of each study. Large studies distribute in the top of the plot, and smaller studies scatter toward the bottom of the plot.
